# Supplementary material for: Using artificial intelligence-based software for an unbiased discrimination of immune cell subtypes in the fracture hematoma and bone marrow of non-osteoporotic and osteoporotic mice
Source: PLoS One. 2025 Apr 29;20(4):e0322542. doi: 10.1371/journal.pone.0322542 (PMC12040195; doi:10.1371/journal.pone.0322542)
Supplement: Attachment — Submitted filename: Response to reviewers.pdf [file pone.0322542.s005.pdf]

universität ulm | DIE DEUTSCHEN UNIVERSITÄTSKLINIKA® | UNIVERSITÄTS KLINIKUM ulm

Institut für Unfallchirurgische Forschung und Biomechanik

Direktorin
Prof. Dr. Anita Ignatius
Helmholtzstraße 14
89081 Ulm (Hausadresse)

Sekretariat
med.biomechanik@uni-ulm.de
T: 0731-500 55301
F: 0731-500 55302
www.biomechanics.de

17.03.25

CLARCERT
Zertifiziertes Qualitätsmanagement
DIN EN ISO 9001:2015

Institut für Unfallchirurgische Forschung und Biomechanik – 89081 Ulm

Mohammed E. Elsalanty
Academic Editor
PLOS ONE

Submission of a revised manuscript

Dear Dr. Elsalanty,

Please find attached our revised manuscript entitled “Using artificial intelligence-based software for an unbiased discrimination of immune cell subtypes in the fracture hematoma and bone marrow of non-osteoporotic and osteoporotic mice”.

We thank the editor for their careful review of our manuscript and the helpful suggestions. We revised the manuscript accordingly. Please see our point-to-point reply attached.

We would greatly appreciate it, if you considered our paper for publication in Plos One.

Kind regards,

M. Haffner-Luntzer

Prof. Dr. Melanie Haffner-Luntzer

UNIVERSITÄTSKLINIKUM ULM Anstalt des Öffentlichen Rechts, Sitz Ulm | UST-ID. Nr. DE147040060
Vorsitzender des Aufsichtsrats: Clemens Benz. Vorstand: Prof. Dr. Udo X. Kaisers (Vorsitzender),
Bettina Rottke (komm., Stellv.), Prof. Dr. Peter Möller, Prof. Dr. Thomas Wirth, N.N.
Sparkasse Ulm IBAN: DE16 6305 0000 0000 1064 78 BIC: SOLADES1ULM

Point-to-point reply

This study involved a post-hoc analysis of flow cytometry data using an AI algorithm. The aim of the study was to further identify sub-classifications of immune cells involved in the early fracture healing, both at the hematoma and the contralateral bone marrow. The premise of the study was quite intriguing and the findings could provide basis for further investigation of key cellular profiles most important for the critical early stage of bone healing. There were a few areas where the study could improve the impact of its findings: 1. Although the use of AI could reduce human bias inherent in the subjective thresholding, AI algorithms are still marred with subjectivity. Up to this point, there still is significant approximations and guesswork built in. Although it would probably be impossible for the investigators to evaluate how consistent the specific logic was across their samples or, equally important, how reproducible it would be for a future study, it would help if they identify the criteria based on which the algorithm made the distinction between subtypes and the weight given to each.

Answer: We thank the editor for this important comment. Indeed, verifying the consistency of an AI-based algorithm is very important. The best way to test for stability is to run the same experiment in duplicates using the subsampling setting while reducing the amount of input data for the algorithms. This approach helps determine how stable the results are and whether there is a threshold at which clusters or populations become undetectable due to excessive downsampling. A recommended strategy is to downsample to one-third of the original data and allow the pipeline to recalculate. Cluster identification is performed using the "PARC" algorithm, an unsupervised learning and open-source algorithm. PARC autonomously detects clusters and is therefore making the distinctions based on the data (see also explanation to the second point). We indeed verified our data by this approach and have now added this information to the manuscript on page 7.

2. To clarify the point above, the authors identified two subpopulations that were most different: AI_subcluster_Bcells_31 and AI_Based_Subcluster_granulocytes_0 and included some cellular features and expression profiles for each. Was these features determined after the AI determination? or were they the only/main criteria used to make the distinction by the algorithm itself? Were there other criteria?

Answer: Supopulation identification is solely performed using the "PARC" algorithm, an unsupervised learning and open-source algorithm. PARC autonomously detects clusters/supopulations and is therefore making the distinctions based on the data only. Once set and defined, the "PARC" algorithm operates independently of user-defined population specifications, ensuring that cluster formation and composition remain unaffected by user decisions (e.g. like population definition criteria). The user is only responsible for defining how the clusters are annotated, determining which subtypes receive specific labels. However, the clusters themselves are computed beforehand and are not influenced by this annotation process. The Cluster Explorer utilizes the median fluorescence intensity (MFI) of each selected cluster (subpopulation) for comparison, highlighting the most significant differences in expression between clusters. It identifies the largest distances between clusters, revealing where the greatest variations in expression occur for a given channel in the compared clusters. These data are partially shown in Fig. 6. As per definition, not only FSC, SSC, DEAD-staining, Ly6G expression and CD11b expression were used by the algorithm to detect subpopulations, but also all other available information. However, since the other markers (CD19, CD3,

CD8, CD4, F4/80) are generally not expressed on neutrophils, emphasis was much less from the algorithm on these markers, this is why we do not display them in Fig. 6. We added this information to the manuscript on page 7.

3. Along the same line, it would be important for future comparisons to include a table where all the identified subpopulations are described in the same manner as the above mentioned two.

Answer: We added a table with the files from the cluster explorer for all subpopulations, please see supplemental table 3.
